# Synthesis, Characterization and Gas Sensing Properties of Ag@α-Fe_2_O_3_ Core–Shell Nanocomposites

**DOI:** 10.3390/nano5020737

**Published:** 2015-05-05

**Authors:** Ali Mirzaei, Kamal Janghorban, Babak Hashemi, Anna Bonavita, Maryam Bonyani, Salvatore Gianluca Leonardi, Giovanni Neri

**Affiliations:** 1 Department of Materials Science and Engineering, Shiraz University, 71946-85115 Shiraz, Iran; E-Mails: alisonmirzaee@yahoo.com (A.M.); janghor@shirazu.ac.ir (K.J.); hashemib@shirazu.ac.ir (B.H.); maryam.bonyani@gmail.com (M.B.); 2 Department of Electronic Engineering, Chemistry and Industrial Engineering, University of Messina, C.da di Dio, 98166 Messina, Italy; E-Mails: abonavita@unime.it (A.B.); leonardis@unime.it (S.G.L.)

**Keywords:** Ag@α-Fe_2_O_3_, core–shell, nanocomposite, synthesis, gas sensor, ethanol

## Abstract

Ag@α-Fe_2_O_3_ nanocomposite having a core–shell structure was synthesized by a two-step reduction-sol gel approach, including Ag nanoparticles synthesis by sodium borohydride as the reducing agent in a first step and the subsequent mixing with a Fe^+3^ sol for α-Fe_2_O_3_ coating. The synthesized Ag@α-Fe_2_O_3_ nanocomposite has been characterized by various techniques, such as SEM, TEM and UV-Vis spectroscopy. The electrical and gas sensing properties of the synthesized composite towards low concentrations of ethanol have been evaluated. The Ag@α-Fe_2_O_3_ nanocomposite showed better sensing characteristics than the pure α-Fe_2_O_3_. The peculiar hierarchical nano-architecture and the chemical and electronic sensitization effect of Ag nanoparticles in Ag@α-Fe_2_O_3_ sensors were postulated to play a key role in modulating gas-sensing properties in comparison to pristine α-Fe_2_O_3_ sensors.

## 1. Introduction

Due to peculiar properties originated from their individual phases, composite materials are of special interest. Among different structures of composites, recently a lot of interest in core–shell nanocomposites, in which a core is coated with a shell, has arisen due to their unique advantages, such as stability, high catalytic activity, controllable compositions/structure, *etc*. and potential applications in fields such as catalysis, optics, biotechnology and gas sensors [1]. For gas sensing application, metal oxide@metal oxide core–shells have attracted great interest because of the different combination of *n*-type and *p*-type metal oxides such as *n*-*n*, *n*-*p* or *p*-*p* can leads to synergetic effects that enhance gas sensing properties. However, recent investigations about metal@metal oxide core–shell as gas sensor have suggested that combination of metal (core) and metal oxide (shell) structure can enhance the sensitivity of gas sensor [2,3]. For example Zhu *et al.* [4] reported an ethanol gas sensor using Ag@TiO_2_ that showed higher sensing properties in comparison with pure TiO_2_. In fact from practical point of view when metal is as a core it not affected by surrounding medium and consequently corrosion or dissolution metal will not occur. Furthermore, because metal is as a core, it will not decrease effective surface area of metal oxide for sensing application. Accordingly, core–shells with metal core and semiconductor shell become increasingly among the strongest candidates as sensor due to their controllable chemical and colloidal stability within the shell and charge transfer between the metal core and semiconductor [5,6].

Among the different combinations of metal@metal oxide core–shell composites, Ag@Fe_2_O_3_ is very interesting: α-Fe_2_O_3_ (hematite) is not only one of the cheapest semiconductor materials (*n*-type, *E_g_* = 2.1 eV) available, but also is one of the most environmental friendly semiconductors. Thanks to its low cost, high resistance to corrosion, and nontoxicity properties, this most stable iron oxide has been traditionally used as catalysts, pigments, electrode materials and gas sensor [7,8,9]. Regarding gas sensor applications, there are many reports about α-Fe_2_O_3_ nanostructures [10,11] and many researchers have tried to enhance gas sensing properties of iron oxide using different strategies such as doping with metals [12], composite with another metal oxide [13] and synthesis of different morphologies [11]. Ag as a core is the cheapest available noble metal and displays excellent thermal conductivity properties, and its electrical conductivity is the best amongst other excellent candidate metals like Cu, Al and Au [14].

Regarding the methods for producing metal@metal oxide core–shell nanoparticles, so far several methods such as precipitation [2,3] and hydrothermal [6], *etc.* have been developed. However, two step reduction-sol gel method is one of the simplest and cheapest procedures for the synthesis of metal@metal oxide core–shell nanocomposites.

To the best of our knowledge, there is no published report about Ag@α-Fe_2_O_3_ core–shell nanocomposites for gas sensing applications. So, aim of the present study is the simple chemical synthesis of Ag@α-Fe_2_O_3_ nanoparticles and investigation of gas sensing properties towards ethanol. Ethanol gas sensors can be applied in many fields, such as the control of fermentation processes, safety testing of food packaging, and can also to monitor drunken driving [15]. A comparative gas sensing study between the Ag@α-Fe_2_O_3_ core–shell and α-Fe_2_O_3_ nanoparticles was then here performed to demonstrate the better gas sensing properties of the core–shell structure.

## 2. Results and Discussion

### 2.1. SEM and TEM Investigations

**Figure 1:**
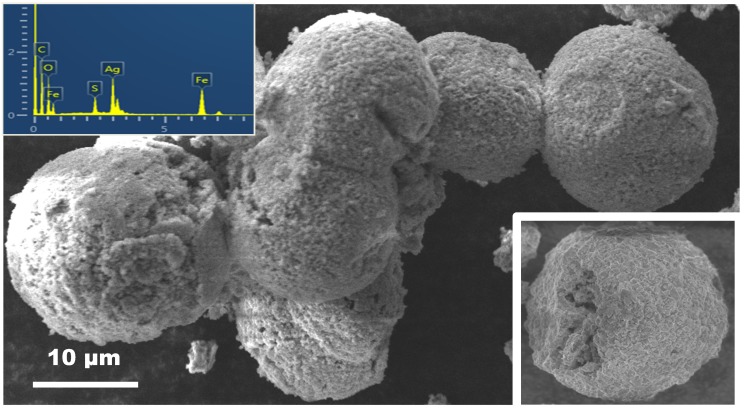
Scanning electron microscopy (SEM) micrograph showing the morphology of Ag@α-Fe_2_O_3_ composite. In the inset are shown one single agglomerate and the energy-dispersive X-ray spectrum (EDX) spectrum.

Figure 1 shows a panoramic view of the morphology of Ag@α-Fe_2_O_3_ composite sample annealed at 600 °C. Large spherical particle agglomerates, having an average size of about 50 μm, have been observed. The inset in Figure 1 reports one such agglomerate, on which is well visible the void inside the structure. Energy-dispersive X-ray spectrum (EDX) confirms that the nanocomposite contains Ag, Fe and O as the main elements. Sulfur, coming from the raw material used as precursors, is also evidenced. The presence of Cu and C in the spectrum is due to the grid used for the scanning electron microscopy (SEM) observation.

**Figure 2:**
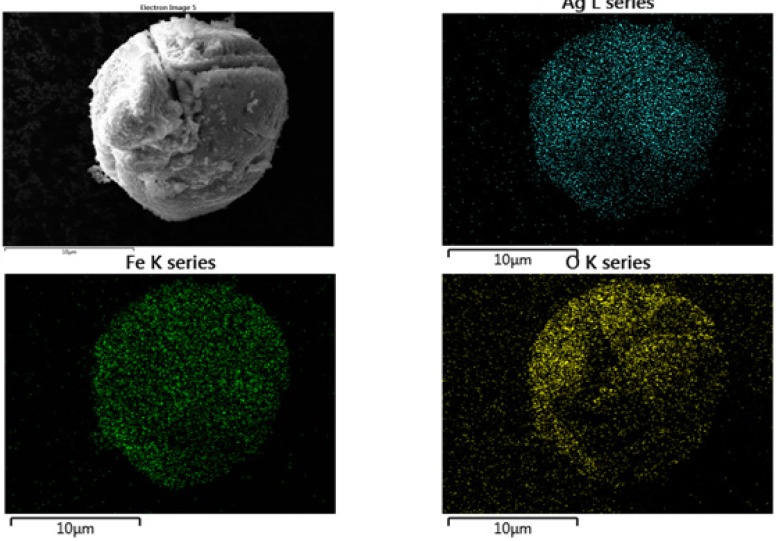
EDX elemental mapping showing the spatial distribution of Ag, Fe and O elements on the surface of the Ag@α-Fe_2_O_3_ nanocomposite agglomerate showed in the SEM image.

To have indications about the distribution of these elements on the sample surface, EDX elemental mapping analysis of the composite has been undertaken. Figure 2 shows the EDX elemental mapping of one particles aggregate, where the X-ray signal coming from Ag, Fe, and O, are represented by blue, green and yellow color, respectively. Data obtained shows the good uniformity regarding the spatial distribution of all these elements in the nanocomposite.

**Figure 3:**
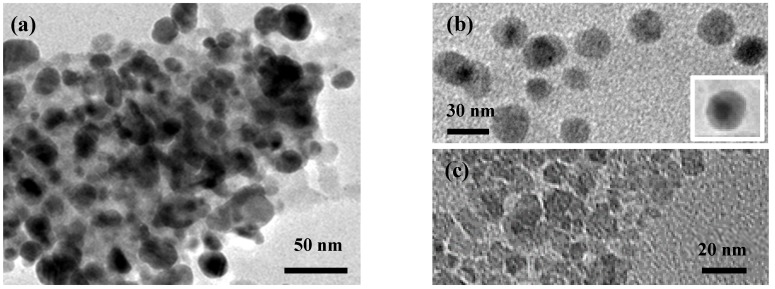
Transmission electron microscopy (TEM) images of: (**a**) and (**b**) Ag@α-Fe_2_O_3_ composite; (**c**) α-Fe_2_O_3_.

Transmission electron microscopy (TEM) analysis has been also performed to investigate the microstructure of the composite sample. Figure 3a reports a TEM image of the composite sample showing one large aggregate of nanoparticles.

In Figure 3b are visible isolated composite nanoparticles showing the core formed by Ag nanoparticles (AgNPs) 10–20 nm in diameter, surrounded by a shell of iron oxide having an average thickness of about 10–20 nm. It is interesting to note that, compared to α-Fe_2_O_3_ sample (see Figure 3c), Ag@α-Fe_2_O_3_ nanoparticles appears to be almost round, suggesting that the presence of Ag NPs hinders the crystallization process of the iron oxide. This is in agreement with XRD pattern of the Ag@α-Fe_2_O_3_ composite (not shown), which highlight the presence of metallic Ag while peaks of α-Fe_2_O_3_ are of very low intensity.

### 2.2. UV-Vis

**Figure 4:**
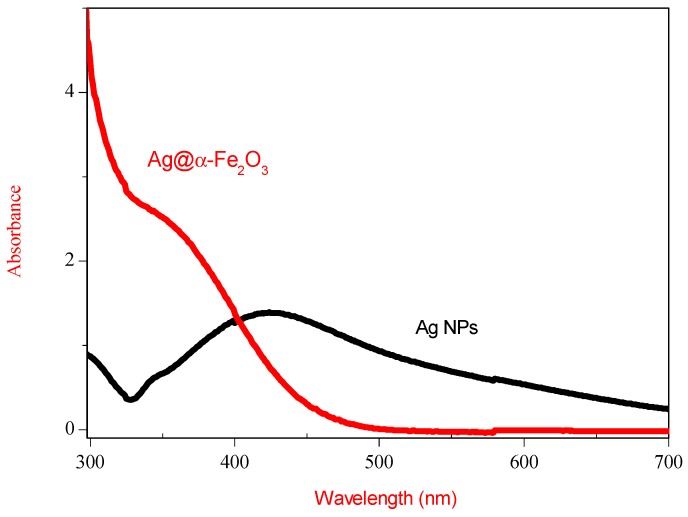
UV-Vis spectra of Ag nanoparticles and Ag@α-Fe_2_O_3_ core–shell nanocomposites.

Optical absorptions spectra of Ag and Ag@α-Fe_2_O_3_ nanoparticles measured as a function of the wavelength are shown in Figure 4. As-synthesized Ag colloid exhibit a absorption peak at 422 nm, a characteristic peak assigned to Ag colloid with diameter <50 nm [16]. After formation of Ag@α-Fe_2_O_3_ core–shell, Ag plasmon peak exhibits a blue shift with simultaneous decrease in peak intensity. According to [17], this shift is due to the scattering effect of Fe_2_O_3_ shell around silver core.

**Figure 5:**
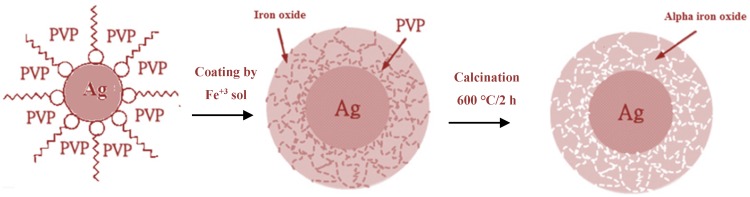
Schematic mechanism for the formation of core@shell structured Ag@α-Fe_2_O_3_.

According to characterization results, formation mechanism for Ag@α-Fe_2_O_3_ core–shell nanoparticles is shown in Figure 5. Upon adding PVP to the AgNO_3_ solution, Ag^+^ nuclei become covered by surfactant, and after adding NaBH_4_, they are reduced to form Ag^0^ nanoparticles. The steric repulsion of PVP inhibits agglomeration of Ag^0^ nanoparticles. When iron precursor is added, it is adsorbed on the surface of Ag NPs and subsequently slowly hydrolyzed, forming a shell around the Ag core. After calcination surfactant molecules are removed, and the shell is thermally decomposed to generate the hematite α-Fe_2_O_3_ shell, producing a uniform core–shell of α-Fe_2_O_3_ nanoparticles.

### 2.3. Sensing Tests

**Figure 6:**
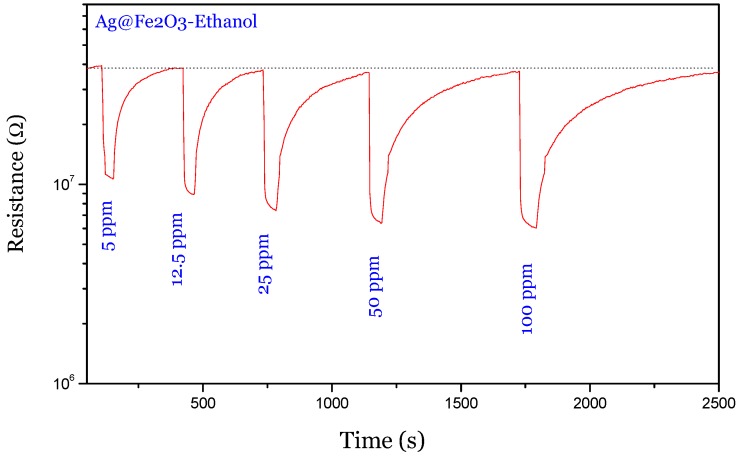
Sensing response of Ag@α-Fe_2_O_3_ towards different concentrations of ethanol vapor at 250 °C.

The synthesized composite with a core–shell structure has been used to fabricate gas sensor devices for detection of low concentrations of ethanol in air. First, a lot of preliminary work has been done to find the optimal operation temperature. The temperature of 250 °C has been chosen on the basis of the better performance obtained for ethanol detection in terms of sensitivity, selectivity and fast response/recovery time. Figure 6 shows the dynamic gas sensing characteristics of Ag@α-Fe_2_O_3_ towards different low concentrations of ethanol in air. The sensor showed the typical *n*-type gas sensing behavior, which can be explained as following. At the beginning of the sensing experiment, the sensor exposed to a dry air atmosphere exhibited a high resistance due to the oxidation reaction between surface electrons and oxygen molecules. After introduction of ethanol vapor into the testing chamber, the baseline resistance was immediately reduced. When the ethanol vapor was cut off from the chamber, the resistance was completely recovered to the original value in base gas. This result indicates that the Ag@α-Fe_2_O_3_ sensor is fully reversible to ethanol.

**Figure 7:**
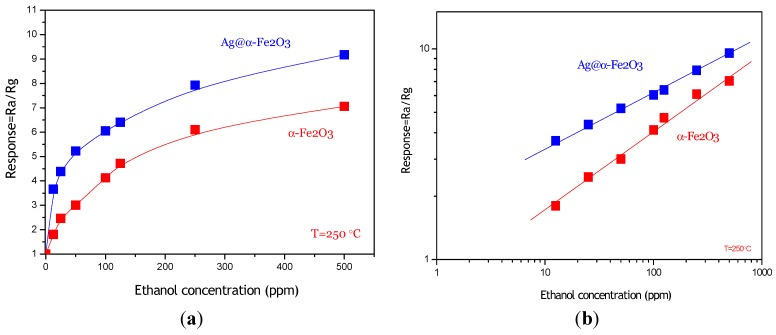
(**a**) Sensing response of Ag@α-Fe_2_O_3_ and α-Fe_2_O_3_ towards different concentrations of ethanol vapor at 250 °C; (**b**) Linear relationship of log S-log C for Ag@α-Fe_2_O_3_ and α-Fe_2_O_3_ sensors.

The calibration curve of the Ag@α-Fe_2_O_3_ sensor to different concentrations of ethanol vapor is shown in Figure 7a. For comparison, in the same graph, the calibration curve related to pristine α-Fe_2_O_3_ sensor is also plotted. It can be clearly evidenced that the composite-based sensor is more sensitive compared to pristine α-Fe_2_O_3_ sensor in the range of concentrations investigated, which is in agreement with results of Zhu *et al.* [4]. As shown in Figure 7b, the response of both sensors has a good linear relationship with the ethanol concentration (12.5–500 ppm range) in logarithmic forms, which is in good agreement with the theory of power laws proposed for semiconductor sensors [18]. The approximate linearity of sensitivity in the broad concentration range is quite suitable for the measurement of ethanol, which could offer wide applications for ethanol detection. Furthermore, it appears that the composite sensor is able to detect sub-ppm concentrations of ethanol with high signal/noise ratio.

**Figure 8:**
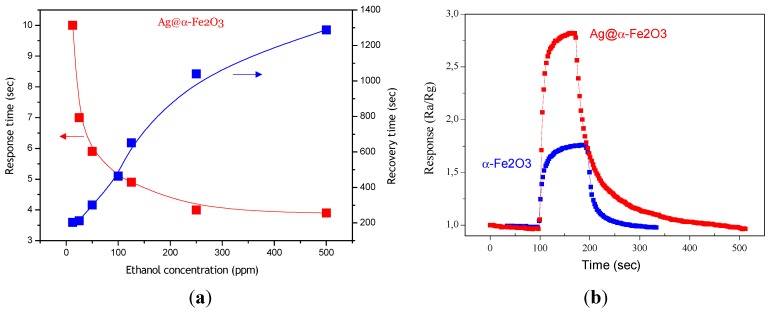
(**a**) Response and recovery times for Ag@α-Fe_2_O_3_ sensor; (**b**) Response of Ag@α-Fe_2_O_3_ and α-Fe_2_O_3_ sensors towards 12.5 ppm ethanol at 250 °C.

Figure 8a shows response and recovery time for Ag@α-Fe_2_O_3_ sensor as a function of ethanol concentration. As it can be seen, with increase of ethanol concentration, response time decreases and recovery time increases. Indeed as ethanol concentration increases more ethanol molecules interact with adsorbed oxygen ions, providing a fast response (shorter time), and obviously they need more time to desorb from surface. The response times are shorter for Ag@α-Fe_2_O_3_ core–shell nanocomposites in comparison with α-Fe_2_O_3_ (Figure 8b). According to the response/recovery time definition, for 100 ppm ethanol response time and recovery time are 5.5 s and 16 s, 453 s and 165.39 s for Ag@α-Fe_2_O_3_ and α-Fe_2_O_3_, respectively. Decrease in response time is probably due to decrease of activation energy of adsorption of ethanol in the presence of silver. However, recovery times are longer for Ag@α-Fe_2_O_3_ nanoparticles. The slow recovery can be explained by the sluggish surface reactions regarding the adsorption, dissociation, and ionization of oxygen [19].

**Figure 9:**
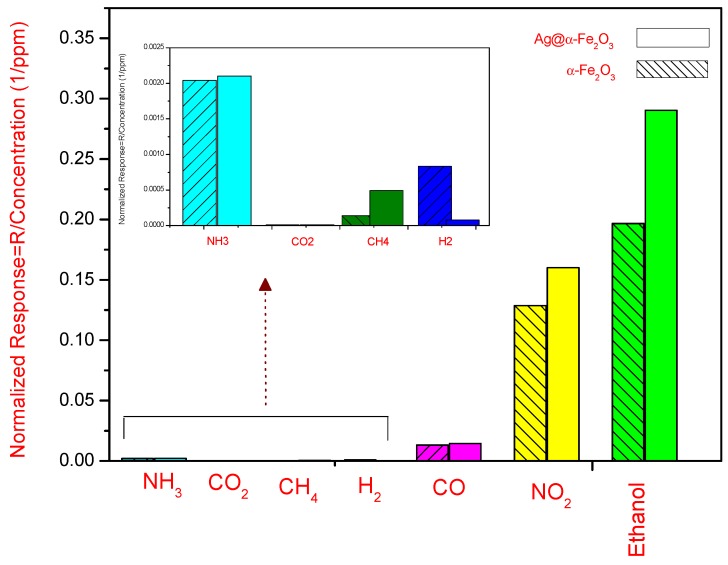
Normalized response of Ag@α-Fe_2_O_3_ and α-Fe_2_O_3_ sensors at 250 °C. In the inset, the response to some reducing gases is magnified.

The normalized response (R/concentration) of both sensors towards different gases such as NO_2_, CO, CO_2_, *etc*. is shown in Figure 9.

It indicates that the sensing response towards ethanol for both sensors is higher than for other reducing gases investigated (especially NH_3_, CO_2_, CH_4_ and H_2_). The sensors show instead a certain sensitivity to NO_2_. However, the magnitude of the normalized response, for ethanol and NO_2_, is 0.2904 and 0.1968, 0.16 and 0.12875 for Ag@α-Fe_2_O_3_ and α-Fe_2_O_3_ sensors, respectively. These values also demonstrated that Ag@α-Fe_2_O_3_ sensor is more selective than α-Fe_2_O_3_ sensor.

### 2.4. Sensing Mechanism

For metal oxide gas sensors, the sensing is based on the change in resistance, which is caused by the adsorption and desorption of ethanol molecules on the surface of sensing films in ethanol atmospheres. When α-Fe_2_O_3_ or Ag@Fe_2_O_3_ sensors are exposed to air, oxygen molecules would be adsorbed on the surface of oxide. The adsorbed oxygen molecules would trap electrons from conduction band of α-Fe_2_O_3_ because of the strong electronegativity of oxygen atom, and produce ionized oxygen species such as ${\text{O}}_{2}^{-}{\text{ O}}^{-}$, O^2−^. The reaction kinetics may be explained by the following reactions [20]:

(1) $${\text{O}}_{2(\text{gas})}\to {\text{O}}_{2(\text{ads})}$$

(2) $${\text{O}}_{2(\text{ads})}+\bar{\text{e}}={\text{O}}_{2(\text{ads})}^{-}\;$$

(3) $${\text{O}}_{2(\text{ads})}^{-}+\bar{\text{e}}=2{\text{O}}_{\left(\text{ads}\right)}^{-}$$

(4) $${\text{O}}_{\left(\text{ads}\right)}^{-}+\bar{\text{e}}={\text{O}}_{\left(\text{ads}\right)}^{2-}$$

The electrons transfer from the conduction band to the chemisorbed oxygen results in the decrease in the electron concentration and an increase in the resistance of α-Fe_2_O_3_ and Ag@α-Fe_2_O_3_. When the α-Fe_2_O_3_ or Ag@α-Fe_2_O_3_ sensor is exposed to ethanol, the ethanol molecules could be chemisorbed on the surfaces of α-Fe_2_O_3_ or Ag@α-Fe_2_O_3_ nanoparticles, and these chemisorbed ethanol molecules would react with the adsorbed oxygen species to form H_2_O and CO_2_. The trapped electrons are then released back to the α-Fe_2_O_3_ or Ag@α-Fe_2_O_3_ nanoparticles, leading to a decreased resistance of the sensor [4]:

(5) $${\text{C}}_{2}{\text{H}}_{5}{\text{OH}}_{\left(\text{ads}\right)}+{\text{O}}_{\left(\text{ads}\right)}^{-}={\text{CH}}_{3}{\text{CHO}}_{\left(\text{ads}\right)}+{\text{H}}_{2}\text{O}+\bar{\text{e}}$$

(6) $${\text{CH}}_{3}{\text{CHO}}_{\left(\text{ads}\right)}+5{\text{O}}_{\left(\text{ads}\right)}^{-}=2{\text{CO}}_{2}+2{\text{H}}_{2}\text{O}+5\bar{\text{e}}$$

(7) $${\text{CH}}_{3}{\text{CHO}}_{\left(\text{ads}\right)}+6{\text{O}}_{\left(\text{ads}\right)}^{2-}=2{\text{CO}}_{2}+3{\text{H}}_{2}\text{O}+12\bar{\text{e}}$$

Accordingly, the magnitude of sensor response depends on two factors: the number of electrons which are captured by adsorbed oxygen molecules from the conduction band and the number of electrons that are released by the ethanol molecules reacting with adsorbed oxygen molecules.

**Figure 10:**
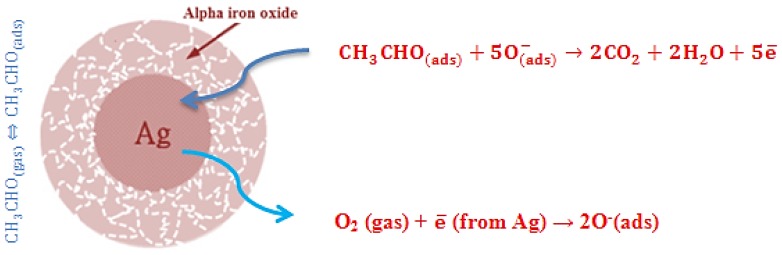
Possible ethanol sensing mechanism by Ag@α-Fe_2_O_3_ core–shell nanocomposites (according to [4,21]).

In case of Ag@α-Fe_2_O_3_, according to [4,21], Ag core has three crucial rules: (i) an electron donates to gaseous oxygen, according to Equations (2)–(4), and consequently accelerate these reactions on the surface; (ii) an electron reservoir releases electrons from surface, according to Equations (5)–(7); and (iii) there is decreased ethanol adsorption and desorption energy on the iron oxide shell. Therefore, in the case of Ag@α-Fe_2_O_3_, sensor response is higher than pure α-Fe_2_O_3_ and the response times of Ag@α-Fe_2_O_3_ are shorter than pristine α-Fe_2_O_3_. A possible sensing mechanism for Ag@α-Fe_2_O_3_ sensor is presented in Figure 10.

In summary, the peculiar hierarchical nano-architecture and the chemical and electronic sensitization effect of Ag nanoparticles in Ag@α-Fe_2_O_3_ sensors are the key factors in enhancing the ethanol response of the nanocomposite-based sensor in comparison to pristine α-Fe_2_O_3_ sensor. The same apply to explain the enhanced selectivity of the Ag@α-Fe_2_O_3_ sensor compared to that of α-Fe_2_O_3_ one. Indeed, Ag NPs likely promote oxygen adsorption/dissociation, thus favoring the reactions involved in the sensing mechanism and resulting in the surface reactivity enhancement for ethanol with respect to other interfering gases.

## 3. Experimental Section

### 3.1. Chemicals

All chemicals used in the experiment were analytic reagent (AR). Silver nitrate (≥99.0% AgNO_3_) as Ag source, sodium borohydride as reducing agent (≥99.0% NaBH_4_), citric acid (>99.0% C_6_H_8_O_7_ × H_2_O) as chelating agent, PEG-4000 as a esterification agent, iron sulfate (≥97% Fe_2_(SO_4_)_3_ × H_2_O) as α-Fe_2_O_3_ source and poly(vinylpyrrolidone) (PVP-M_W_ ~ 1300000) as stabilization agent were obtained from Sigma-Aldrich. Redistilled water was used to prepare all of solutions.

### 3.2. Ag Nanoparticle Synthesis

The solutions of PVP and AgNO_3_ were prepared by dissolving appropriate amounts of PVP and AgNO_3_ in redistilled water in separate well-cleaned dry beakers at room temperature. Then PVP solution and AgNO_3_ solution were mixed and magnetically stirred for 60 min to form Ag^+^-PVP solution. In a separate beaker, the NaBH_4_ solution was prepared by dissolving the required amount in distilled water. Ice bath was used to slow down the reaction and give better control over final particle size/shape. Then NaBH_4_ solution was added dropwise, (about 1 drop/second, until it is all used up) to Ag^+^-PVP solution under vigorous stirring and solution stirred for another 10 min. Upon sodium borohydride addition, the color of the solution slowly turned into pale yellow, indicating the reduction of the Ag^+^ ions to metallic silver.

### 3.3. Ag@α-Fe_2_O_3_ Nanoparticle Synthesis

**Figure 11:**
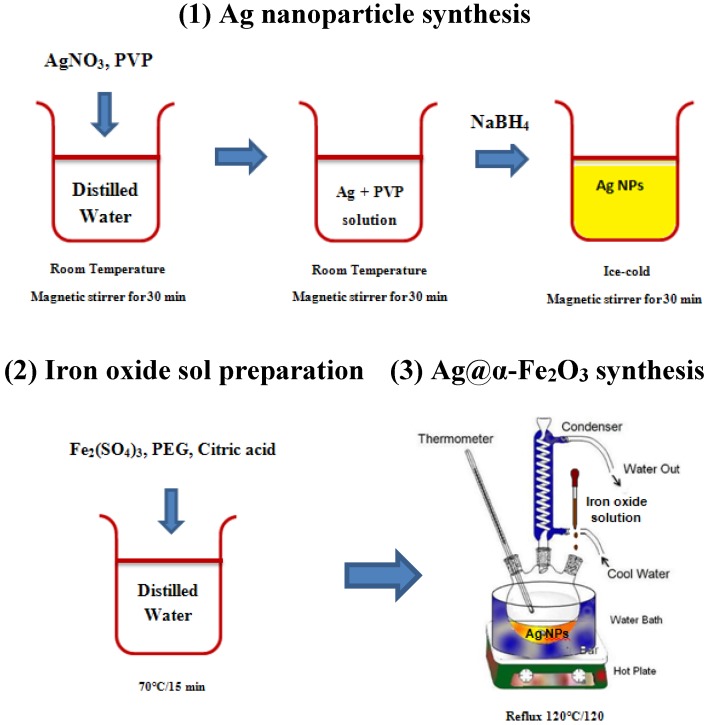
Schematic representation of synthesis procedure of Ag@Fe_2_O_3_ core-shell nanoparticles.

Core–shell Ag@α-Fe_2_O_3_ nanocomposites were synthesized using a sol-gel coating process as follow: to coat the silver nanoparticles, an Fe^+3^ sol was prepared by Pechini sol-gel method using iron sulfate (III) as an Fe^+3^ source, citric acid as a chelating agent and PEG-6000 as a esterification agent (details can be found in the [22]). The Fe^+3^ sol was added drop by drop to the solution of silver colloids and the solution was stirred for 15 min. Finally, the mixture was refluxed for 2 h under moderate stirring at 120 °C and the core–shell solution of Ag@α-Fe_2_O_3_ was prepared. Figure 11, reports a schematic picture of the synthesis procedure. For comparison, pure α-Fe_2_O_3_ nanoparticles were synthesized by the same Pechini sol-gel method according to [22].

### 3.4. Characterization

Morphological analysis was carried out by SEM using a ZEISS 1540XB FE SEM instruments (Zeiss, Germany) equipped with an EDX detector. Particle size and shell formation studies of synthesized core–shells were performed with a JEOL JEM 2010 electron microscope (LaB6 electron gun), (JEOL, Tokyo, Japan) operating at 200 kV and equipped with a Gatan 794 Multi-Scan CCD camera.

Optical absorbance of silver nanoparticles as well as Ag@α-Fe_2_O_3_ core–shells were recorded using a UV–Vis spectrophotometer (Evolution 500, Thermo Electron Corp., USA,) as a function of wavelength in the range from 300 to 700 nm.

### 3.5. Gas Sensing Measurements

To measure the sensor response of the core–shell nanoparticles for different gases, a sensor device was prepared as follows: the synthesized Ag@α-Fe_2_O_3_ core–shell as well as α-Fe_2_O_3_ nanoparticles was drop coated on the cleaned Al_2_O_3_ substrate (6 mm × 3 mm sized) with interdigitated platinum electrodes and a Pt heater located on the back. Before sensing tests, the sensor was conditioned in air for 2 h at 300 °C. The change in resistance of the device due to the presence of different gases was measured. The sensor was tested in the temperature range from 225 to 400 °C at various concentrations under a synthetic, dry air stream of 100 sccm by collecting the resistance data in the four-point mode. The gas sensing was performed by injecting pulses of the analyte from certified bottles. The gas response, *S*, is defined as *S* = *R*_air_/*R*_gas_ for reducing gases and *S* = *R*_gas_/*R*_air_ for oxidizing gases, where *R*_air_ is the baseline resistance in air and *R*_gas_ is the electrical resistance of the sensor in the present of gas.

## 4. Conclusions

In this study, Ag@α-Fe_2_O_3_ core–shell nanoparticles were successfully synthesized using a simple two step chemical route. Characterization techniques confirmed the core–shell structure of Ag@α-Fe_2_O_3_. According to TEM micrographs, the silver core with an average of 20 nm was covered by a porous shell of Fe_2_O_3_ with thickness of 20 nm. As a very cheap gas sensor, the experiments on the gas sensing properties showed that the Ag@α-Fe_2_O_3_ core–shell nanoparticles have better sensitivity to ethanol than α-Fe_2_O_3_ nanoparticles. Moreover it showed negligible response towards other gases, *i.e.*, high selectivity. In conclusion the silver@iron oxide core–shell nanocomposite prepared by the simple two step chemical route have strong potential as a ethanol sensing material with high selectivity.
